# Adenoid cystic carcinoma of the breast: A case report and literature review

**DOI:** 10.3892/ol.2014.1945

**Published:** 2014-03-06

**Authors:** EMINE CANYILMAZ, GONCA HANEDAN USLU, YAHYAHAN MEMİŞ, ZÜMRÜT BAHAT, KADRIYE YILDIZ, ADNAN YONEY

**Affiliations:** 1 Department of Radiation Oncology, Faculty of Medicine, Karadeniz Technical University, Trabzon, Turkey; 2 Department of Radiation Oncology, Kanuni Research and Education Hospital, Trabzon, Turkey; 3 Department of Pathology, Faculty of Medicine, Karadeniz Technical University, Trabzon, Turkey

**Keywords:** breast cancer, adenoid cystic carcinoma, radiotherapy, adjuvant therapy

## Abstract

Adenoid cystic carcinoma (ACC) is a rare malignant tumor of the breast that occurs in <0.1% of all patients diagnosed with breast cancer. The mean patient age at the time of diagnosis is 50–60 years. Typically, the tumor presents as a subareolar mass or as pain in the breast. While the radiological appearances of ACC are generally non-specific, the diagnosis can be made on fine-needle aspiration cytology. In the present study, a 58-year-old female patient was admitted to the Department of Radiation Oncology (Karadeniz Technical University, Faculty of Medicine, Trabzon, Turkey) with complaints of pain in the upper outer quadrant of the right breast. An excision biopsy of a lump in the upper outer quadrant revealed ACC, and perineural invasion was present. Subsequently, the patient underwent breast conservation surgery and sentinel lymph node dissection. Pathology from the second surgery depicted ACC in the form of microscopic foci around the initial surgical cavity, with two reactive sentinel lymph nodes and the closest negative margin at 2 mm. The patient was treated with radiotherapy following the surgery. No recurrence and metastasis were found after 20 months of follow-up. In conclusion, mammary ACC is a rare malignant neoplasm of the breast. Although surgery is the main treatment, the optimal adjuvant treatment of ACC of the breast has not yet been determined due to its low incidence.

## Introduction

Adenoid cystic carcinomas (ACCs) are rare malignant tumors of the breast. Although most frequently noted in the salivary glands, ACCs are common in the uterine cervix, skin, lungs, kidneys, esophagus and prostate. These tumors occur in <0.1% of all patients diagnosed with breast cancer. The age distribution is from 19–97 years, and the condition is more common in the 50 to 60-year-old age group (1–3). Typically, the tumors present as a subareolar mass or as pain in the breast (4,5). The involvement of lymph nodes and distant metastasis are extremely rare. ACC of the breast shares the same histological characteristics with ACC of the salivary gland. The prognosis of ACC of the breast is improved in comparison to other pathological types of breast cancer and ACC of the salivary gland (1,6). High survival rates following mastectomy or breast protective surgery have been reported previously (7).

ACC of the breast has a biphasic pattern. Histologically, it consists of small basaloid cells with a solid cribriform pattern or epithelial cells with a tubular growth pattern. Although the exact origins remain unknown, it is estimated that these tumors originate from the ductal epithelium or myoepithelium. The presence of estrogen and progesterone receptors tends to be negative in these tumors (1). ACC has an excellent prognosis, a low local recurrence and a rare distant metastasis (8,9).

The aim of the present study was to report the case of a patient who was admitted to the the Department of Radiation Oncology (Karadeniz Technical University, Faculty of Medicine, Trabzon, Turkey) with a diagnosis of ACC of the breast, and review the clinical presentation in light of existing literature. Patient provided written informed consent.

## Case report

**Figure 1:**
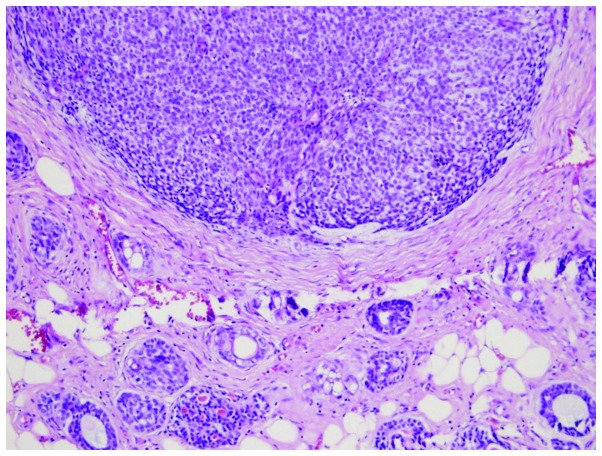
Cribriform growth pattern and neural invasion (HE; magnification, ×100). HE, hematoxylin and eosin.

**Figure 2:**
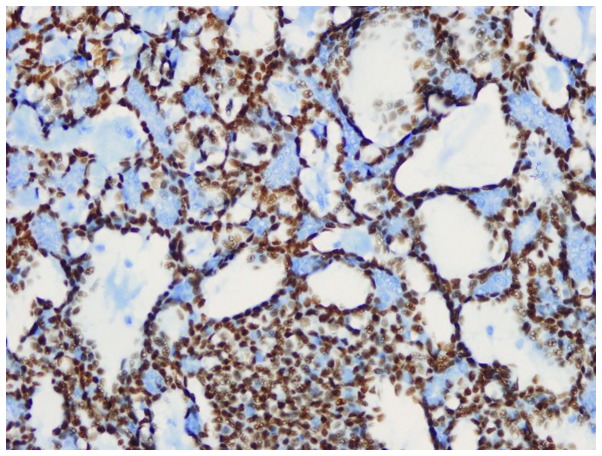
Positive immunohistochemical results for p63 (magnification, ×400).

A 58-year-old postmenopausal patient was admitted to the Department of General Surgery (Karadeniz Technical University, Faculty of Medicine, Trabzon, Turkey) with complaints of pain in the outer quadrant of the right breast. The patient has two children and nothing particularly noteworthy in the personal medical and family histories. The family history was negative for breast cancer and the patient did not smoke or consume alcohol. The breast examination revealed a lump under the upper outer quadrant of the right breast, ~1 cm in diameter. The lump was tough and mobile, however, there was no erythema, ecchymosis, skin ulceration or dimpling identified. No axillary lymphadenopathy was detected and there were no positive findings in the laboratory examinations. On mammography, the patient was noted to have a dense mass with spicular extensions in the upper outer quadrant of the right breast. The mass was reported with a Breast Imaging-Reporting and Data System score of 4, and the lesion was therefore removed by excisional biopsy. The pathology report demonstrated 1×1-cm and 1×0.5-cm ACCs in the form of two close foci, with <30% solid pattern and positive results for cluster of differentiation 56 (CD56) focal immunoreactivity, smooth muscle actin, CD117, high molecular weight keratin and estrogen receptor (2%). The ACCs also had a Ki-67 score of 30%, diffuse nuclear p53 staining, perineural invasion and positive surgical margins. The biopsy specimen was negative for human epidermal growth factor receptor 2 (Her-2/neu), chromogranine, lymphovascular invasion, synaptophysin and the progesterone receptor (Figs. 1 and 2).

The pre-operative abdominal and thoracic tomography showed no distant metastasis. Subsequently, the patient underwent breast conservation surgery and sentinel lymph node dissection. The pathology from the second surgery depicted ACC in the form of a microscopic foci around the initial surgical cavity, with two reactive sentinel lymph nodes and the closest negative margin at 2 mm. Therefore, the patient was treated with a dose of 50 Gy, with a fraction of 2 Gy on a daily basis from two opposing parallel tangential fields post-operatively, using 6-MV photon beams. Following whole breast radiotherapy, a 10-Gy boost on the tumor bed with 12-MeV electron beams was also delivered. Finally, 10 mg tamoxifen was administered to the patient twice daily for 20 months following radiotherapy. No recurrence and metastasis were identified in the patient at month 20 of the follow-up period.

## Discussion

ACC occurs in one in 1 million females every year (7) and was initially described as a cylindroma by Billroth in 1856 (10). Breast ACC was first described by Geschickter in 1945 (11). ACC has three varied growth patterns: Glandular, tubular and solid. Ro *et al* (12) divided the disease into three categories depending on the degree of solid structure in the tumor. Based on this categorization, the grade of the tumor increases with an increased rate of solid element: Grade 1, numerous glands and cystic components, without solid components; grade 2, <30% solid components; and grade 3, >30% solid components. According to this grading system, local excision is recommended for grade 1, simple mastectomy is recommended for grade 2, and mastectomy and axillary dissection is recommended for grade 3. In the present case, the patient underwent breast conservation surgery and sentinel lymph node dissection due to the presence of high-grade disease.

ACCs are commonly detected in postmenopausal females and in the geriatric population (1). However, there are isolated case studies of ACCs occurring in males and children reported in the literature (13). The left and right breasts are equally affected and there is no tendency for the occurrence to be bilateral. ACC is frequently localized in the subareolar upper outer quadrant of the breast and is generally multifocal. Although the tumor size is usually 2–3 cm, the existing literature contains cases with a tumor size of 15 cm. The most frequent symptoms at presentation include the finding of a well-circumscribed palpable mass in the breast, pain in the breast and nipple retraction (1). The postmenopausal patient of the present study complained of breast pain in concordance with the literature, and the tumor, again in agreement with the literature, was localized in the upper outer quadrant and was multifocal.

Perineural invasion is common in ACCs of the salivary gland and this is believed to be the underlying cause for the symptom of pain in these patients. However, perineural invasion is extremely rare in ACCs of the breast (14). In the present case, perineural invasion was present. By mammography, the tumor is characterized as a well-circumscribed lobulated mass that presents with extremely rare microcalcifications, and is associated with hypoechoic lesions on ultrasonography. ACC is generally negative for the estrogen and progesterone receptors and Cerb-B2. Notably, although it has a triple-negative pattern, it does not clinically behave like a triple-negative breast tumor (15). This is explained by the downregulation of genes involved in migration, proliferation and the immune response (1). In the present case, the progesterone receptor and Cerb-B2 were negative, and the estrogen receptor was positive at 2%, which is in agreement with the existing literature.

There is no consensus regarding the optimal treatment of ACCs of the breast due to the rare occurrence of these tumors. Surgical approaches range from local excision to mastectomy (14). Recurrence rates ranging from 6–37% were reported following local excision (16–17). Since extremely few recurrences were reported following mastectomy, numerous clinicians recommend mastectomy for a diagnosis of ACC of the breast. However, there is no randomized controlled trial comparing breast conserving surgery to mastectomy. Recent studies, however, have begun to report higher rates of lumpectomy (18), although, no information about surgical margins has been reported in the majority of patients. The role of radiotherapy following breast conservation surgery remains unclear. Data for the role of radiotherapy in female breast ACCs are limited. There are few studies containing a substantial number of patients receiving adjuvant radiotherapy (12). Arpino *et al* (19) reported that 14 out of 182 patients in a series experienced local recurrence and that 78% of them developed recurrence following local excision. None of these cases received radiotherapy. It was argued that no recurrence occurred in any of the 22% of patients who received radiotherapy in the series, therefore, mastectomy should be avoided. In a study by the Rare Cancer Network (RCN), 66% of 61 patients received post-operative radiotherapy, which was associated with an improvement in local regional control by 12% in five years (95% for the group with post-operative radiotherapy vs. 83% for the group without radiotherapy). Based on the findings of the study, it was recommended that radiotherapy should be performed following lumpectomy irrespective of surgical margins (18). Previously, 376 patients with ACC of the breast were identified by the Surveillance, Epidemiology, and End Results database. In total, 60% of these patients were treated with lumpectomy and 40% underwent mastectomy. Radiotherapy was found to be a strong prognostic factor for overall and cause-specific survival (20). In the present case, breast conservation surgery was performed followed by radiotherapy in the post-operative period.

Axillary lymph node involvement is extremely rare, occurring in an average of 0–2% of ACCs of the breast (1). In a review containing 182 patients who underwent axillary dissection, lymph node metastasis were reported in only 4 patients (17). In a study by the RCN, which monitored 61 patients, an axillary dissection was performed in 41 patients (67%), while a sentinel lymph node biopsy was carried out in 10 patients (16%), all of whom revealed negative lymph nodes. An average 79-month follow-up of these patients showed that there were no axillary supraclavicular fossae or internal mammary chain metastases (18). Therefore, axillary dissection should not be clinically performed except in the presence of nodal metastasis. Additionally, sentinel lymph node sampling can be performed if the tumor is >3 cm, has a high grade or contains other invasive types of breast cancer (1). In the present case, a sentinel lymph node dissection was performed, as the tumor was of a high grade and a sentinel lymph node was found to be negative.

The role of adjuvant therapy and hormonal therapy is controversial in patients with ACC of the breast. The presence of a tumor with good biological characteristics and the absence of a predisposition to distant metastasis increase the importance of local disease control. Arpino *et al* (19) and McClenathan and de la Roza (20) reported that treatments with adjuvant chemotherapy and hormonal therapy do not increase the survival rates of patients. Similarly, it was reported that systemic therapy is a controversial contribution to survival (7). Tamoxifen was administered following radiotherapy and no chemotherapy was performed in the present case since the estrogen receptor was 2% positive.

The 5-year survival rate for ACCs of the breast is reported to be 85–90%, with a 100% disease-free survival rate (2). Despite these results, ACC is reported to present with local recurrences and distant metastases. The most common site for distant metastasis is the lung followed by the liver, kidneys and brain (21).

In conclusion, ACCs of the breast are extremely rare neoplasms of the breast and have an extremely good prognosis. Although no consensus exists regarding the optimal treatment, breast conservative surgical and radiotherapy are recommended. The affected patients require close follow-up due to the rare, but possible, occurrence of distant metastasis.
